# *Ginkgo biloba* Sex Identification Methods Using Hyperspectral Imaging and Machine Learning

**DOI:** 10.3390/plants13111501

**Published:** 2024-05-29

**Authors:** Mengyuan Chen, Chenfeng Lin, Yongqi Sun, Rui Yang, Xiangyu Lu, Weidong Lou, Xunfei Deng, Yunpeng Zhao, Fei Liu

**Affiliations:** 1 College of Biosystems Engineering and Food Science, Zhejiang University, Hangzhou 310058, China; mychen_1998@zju.edu.cn (M.C.); ryang@zju.edu.cn (R.Y.); luxyzju@zju.edu.cn (X.L.); 2 Systematic & Evolutionary Botany and Biodiversity Group, MOE Key Laboratory of Biosystem Homeostasis and Protection, College of Life Sciences, Zhejiang University, Hangzhou 310058, China; 12107151@zju.edu.cn; 3 Institute of Crop Science, College of Agriculture & Biotechnology, Zhejiang University, Hangzhou 310058, China; 12116016@zju.edu.cn; 4 Institute of Digital Agriculture, Zhejiang Academy of Agricultural Sciences, Hangzhou 310021, China; louwd@zaas.ac.cn (W.L.); dengxf@zaas.ac.cn (X.D.)

**Keywords:** *Ginkgo biloba*, sex identification, leaf morphology, hyperspectral imaging, machine learning

## Abstract

*Ginkgo biloba* L. is a rare dioecious species that is valued for its diverse applications and is cultivated globally. This study aimed to develop a rapid and effective method for determining the sex of a *Ginkgo biloba*. Green and yellow leaves representing annual growth stages were scanned with a hyperspectral imager, and classification models for RGB images, spectral features, and a fusion of spectral and image features were established. Initially, a ResNet101 model classified the RGB dataset using the proportional scaling–background expansion preprocessing method, achieving an accuracy of 90.27%. Further, machine learning algorithms like support vector machine (SVM), linear discriminant analysis (LDA), and subspace discriminant analysis (SDA) were applied. Optimal results were achieved with SVM and SDA in the green leaf stage and LDA in the yellow leaf stage, with prediction accuracies of 87.35% and 98.85%, respectively. To fully utilize the optimal model, a two-stage Period-Predetermined (PP) method was proposed, and a fusion dataset was built using the spectral and image features. The overall accuracy for the prediction set was as high as 96.30%. This is the first study to establish a standard technique framework for Ginkgo sex classification using hyperspectral imaging, offering an efficient tool for industrial and ecological applications and the potential for classifying other dioecious plants.

## 1. Introduction

*Ginkgo biloba* L., commonly known as the maidenhair tree, is often celebrated as a “living fossil” and is possibly the oldest known dioecious gymnosperm [1,2]. It has been commonly planted worldwide in cities and towns as a landscape tree and as an industrial plant that produces the leaf extract EGb 761 and edible and medicinal nuts (seeds). Male and female ginkgo trees are differentially preferred in applications. Females are not favored due to their odorous seeds, shedding in fall. Males are more popular in landscape greening because they are taller trees with more compact crowns and longer leaf periods [3]. Contrastingly, females exceed males when people harvest seeds for food or traditional medicine in East Asia [4]. Also, females contain higher contents of flavonoids and terpene trilactones than males, making them more valuable in leaf production for the pharmaceutical industry. Therefore, effective sex identification techniques are crucial for ginkgo trees at multiple developmental stages.

The simplest and most intuitive method to distinguish the sexes of plants is based on their morphological characteristics. Previous studies reported the differences between the two sexes of ginkgo in their reproductive organs (cones) [5,6], vegetative organs [3,7], and phenology [8,9]. However, ginkgo trees have juvenile periods lasting two decades in which they produce no cones. Also, the other morphological methods have poor accuracy and reliability and are highly dependent on experience. The lack of clear standards for the digitization and mapping of morphology has created a signification disparity between shape and quality indices, often leading to the misinterpretation of results [10]. Therefore, the reliability of current methods of morphological identification is highly insufficient.

To achieve satisfactory identification accuracy, a number of molecular markers were developed for the specific identification of ginkgo sexes [11], such as random amplified polymorphic DNA (RAPD) [12] and sequence-characterized amplified region (SCAR) [13]. Notably, a male-specific marker was proposed based on 2.7 Mb sequences specific to the Y chromosome, realizing accurate and reliable identification [14]. However, the weakness of molecular approaches lies in their higher costs in terms of time and money [15].

Meanwhile, diverse analytical methods have been extensively studied based on physiological and biochemical associations with the sexes of ginkgo [11,15,16,17]. Males showed higher contents of quercetin and bilobalide using high-performance liquid chromatography (HPLC) [11] and greater peroxidase activity using electrochemical techniques [15], which were used to distinguish both sexes. Unfortunately, these methods also require long periods for sample preparation and testing.

Recent advancements in spectroscopic techniques have demonstrated significant potential to revolutionize agricultural and forestry practices. A Raman spectrometer was used to determine the sexes of mature Palmer amaranth leaves [18]. Near-infrared reflectance spectroscopy (NIRS), Fourier transform infrared attenuated total reflectance (FTIR/ATR), and nuclear magnetic resonance (NMR) spectroscopy were employed for sex differentiation in immature date palm leaves [19]. Hyperspectral techniques have been shown to be capable of characterizing material contents like peroxidase [20], water [21], chlorophyll [22], and lignin [23] within plants. The spectral absorption bands in the wavelength range of 400–1200 nm are associated with multiple overtones and combinations of the fundamental vibrations of chemical bonds between light atoms [24]. The shapes of spectra obtained from samples are the result of several interactions between radiation and water; organic molecules such as proteins, carbohydrates, and fats; and low-concentration constituents like vitamins and minerals [25]. It has been documented that there are differences in the contents of chlorophyll [26], peroxidase [15], flavonoids [11], lactones [11], and amino acids [16] between the leaves of male and female ginkgo trees. Hence, employing hyperspectral imaging technology as a means to identify the sex of a *Ginkgo biloba* represents a viable technical approach.

Since hyperspectral data analysis is complex, especially when image features are fused, to make sense of these high-dimensional and multi-variate datasets, machine learning methods have been utilized for classification. A U-net network [27], EfficientNet [28], ResNet101 [29], NASNet [30,31], Shufflenet [32], Inception [33], and a deep convolutional neural network (deep CNN) [34] have proved to be effective approaches to solving the problem of image classification, which lacks quantitative criteria. Hu et al. [35] used deep learning techniques to identify the wave sizes caused by fish feeding to guide feeding. It was difficult for the human eye to make a quantitative assessment of wave size, but deep learning could learn high-dimensional features and finally achieved an accuracy of 93.2%. In addition, classical partial least squares discriminant analysis (PLS-DA) [36], linear discriminant analysis (LDA) [37], and subspace discriminant analysis (SDA) [38], as well as support vector machine (SVM) [39,40], are the most widely used machine learning techniques in hyperspectral studies.

Hyperspectral imaging technology has been applied to assess the chemical compositions of plants non-destructively and quickly [41], which can meet the requirements of a real-time, convenient, low-cost, and large-volume technique. Therefore, this study aimed to explore the feasibility of identifying the sexes of *Ginkgo biloba* L. The specific objectives were as follows: (1) to identify ginkgo sexes based on RGB images combined with deep learning; (2) to explore the spectral differences between leaves of males and females to establish an identification model; and (3) to explore the effect of the fusion of spectral and image information on identification accuracy.

## 2. Materials and Methods

### 2.1. Data Acquisition

**Figure 1:**
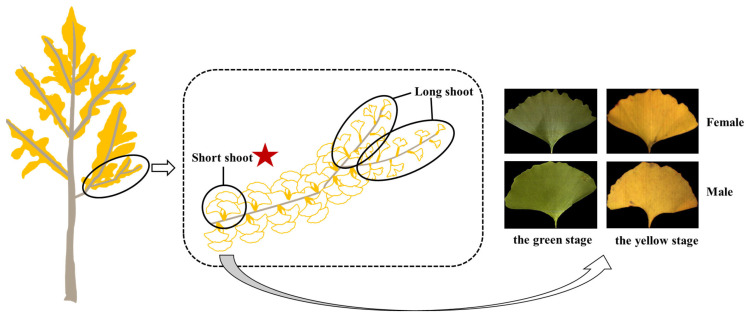
*Ginkgo biloba* leaf collection.

Our study site was located at the Zijingang Campus of Zhejiang University (30°17′48″ N, 120°5′7″ E), Hangzhou, Zhejiang Province, China, where artificially planted ginkgo trees were geo-tagged with sex information based on field observations from long-term phenological monitoring [42]. *Ginkgo biloba* has two leaf types that differ in their morphologies. The short-shoot varieties bear many leaves, which are undivided or slightly bilobed and appear in early spring from overwintering buds, while the leaves of long-shoot varieties are smaller and are deeply divided into two or more lobes. Generally speaking, the shapes of leaves on short branches are consistent, while the shapes of leaves on long branches are easily affected by the growth state [3]. In order to avoid the influence of the development status and environmental conditions on the results of male and female classification, *Ginkgo biloba* leaves were randomly selected from short branches of healthy ginkgo trees at 8 a.m. to 9 a.m., with 10 leaves collected from each ginkgo tree, as shown in Figure 1. The diameter at breast height (DBH) of sample trees ranged between 15.1 and 19.7 cm and showed no significant difference between trees of the two sexes. The collected leaves were stored in a cooler at 4 °C and transported to our laboratory in one hour. After collection, they were inspected to remove leaves with wormholes, defects, or bends. Ginkgo leaves exhibit roughly two states throughout the year, from green to yellow. A total of 1271 green leaves were collected on 11 May and 2 July 2022, including 635 male leaves and 636 female leaves from 89 male and 91 female ginkgo trees. A total of 1306 yellow leaves were collected on 2 November and 29 November 2022, including 652 leaves from 94 male trees and 654 leaves from 92 female trees. 

The hyperspectral instrument selected for this experiment was a high-precision VNIR hyperspectral imager (Pika XC2, Resonon Corporation, Bozeman, MT, USA). A linear push-sweep scanning mode was applied with a spectral coverage wavelength range of 400 to ~1000 nm, a spectral resolution of 2.3 nm, and a spectral channel count of 231. The hyperspectral imaging system was preheated before data acquisition to eliminate the influence of limit drift on the quality of the acquired images.

The leaves were placed without any special treatment on a black blotting cloth for data acquisition, minimizing any background interference. The leaves were positioned uniformly to maintain a consistent distance and angle relative to the hyperspectral imager. The experimental instrument parameters were set as follows: a frame rate of 30 fps, an exposure time of 26 ms, and a scan speed of 4.897 mm/s. Additionally, the hyperspectral data were collected in a dark room to avoid the influence of ambient light, and measures were taken to control and monitor light variations throughout the imaging process to ensure the stability of the acquired hyperspectral data.

### 2.2. Sex Identification Method

**Figure 2:**
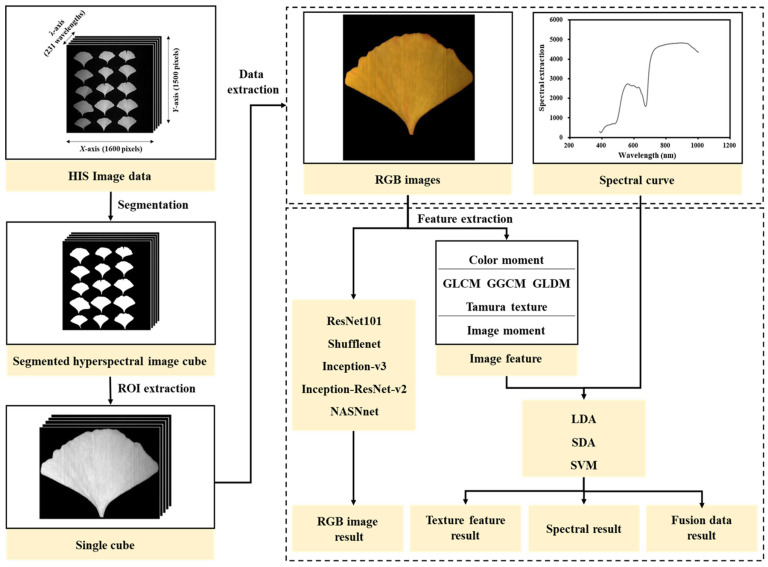
Hyperspectral procedures for sex identification of *Ginkgo biloba* leaves.

The hyperspectral data were composed of two-dimensional geometric space information and one-dimensional spectral information about the measured object. In order to make full use of the data, image data, spectral data, and image features were analyzed. These procedures are shown in Figure 2.

#### 2.2.1. Deep Learning Model

**Figure 3:**
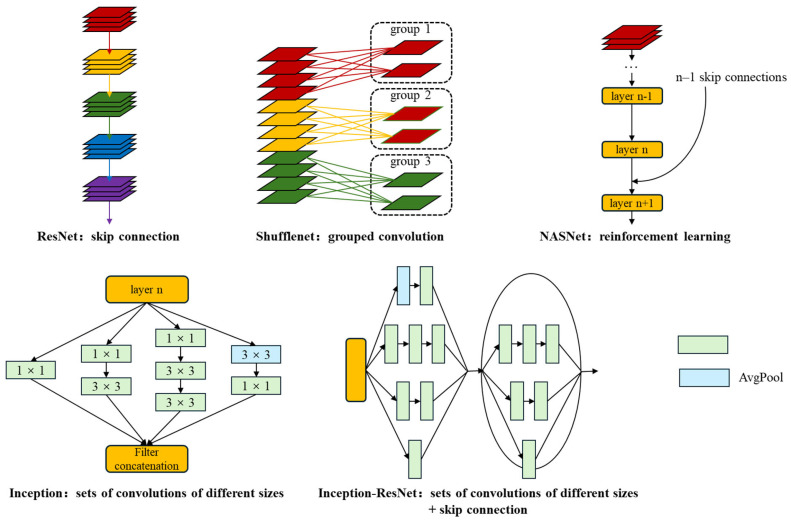
Characteristics of the deep learning network frameworks used.

The aim of this study was to accurately and quickly discriminate the sexes of *Ginkgo biloba* using leaves. The rationale for selecting these specific models is based on their demonstrated performance on large-scale datasets like ImageNet and their varied architectural innovations. ResNet101, Shufflenet, NASNet, Inception-v3, and Inception-ResNet-v2 were selected for this task from the common deep learning models based on two indices: their top-5 error rates on the ImageNet dataset and their reference numbers. Their characteristics are shown in Figure 3.

ResNet101 [43] is a member of the ResNet family of models, which use skip connections to allow a network to learn residual representations of input data, and has been shown to allow the training of very deep neural networks with minimal performance degradation. Shufflenet [44] is a convolutional neural network architecture designed for efficient computation in mobile devices and other resource-constrained environments. It uses channel transformation; i.e., the input channels are divided into groups via convolution operations, the channels within each group are shuffled, and then each group is convolved separately. This allows the network to capture different features while minimizing the numbers of parameters and computations. NASNet [45] is a convolutional neural network designed using reinforcement learning techniques. The NASNet architecture consists of a number of building blocks, each of which can be configured in different ways. The reinforcement learning algorithm searches through the space of possible configurations to find the optimal set of building blocks and their connections. Inception-v3 [46] uses “inception modules”, which are sets of convolutions of different sizes and pooling operations that are performed in parallel, allowing the network to capture information at multiple scales and resolutions, and batch normalization, which helps to reduce overfitting by normalizing the input to each layer of the network. In addition, Inception-v3 uses an auxiliary classifier during training, which helps to prevent the vanishing gradient problem by providing additional supervision to the network. Inception-ResNet-v2 [47] builds on the Inception architecture by adding residual connections between Inception modules. These connections allow for easier training of very deep networks by mitigating the vanishing gradient problem. In addition, Inception-ResNet-v2 also includes label smoothing, which helps prevent overfitting by reducing the network’s confidence in incorrect labels during training, and factorized convolution, which can be more computationally efficient and can lead to better results.

#### 2.2.2. One-Dimensional Data Modeling Methods

LDA, SVM, and SDA were selected to deal with the sex discrimination problem of *Ginkgo biloba*. LDA [48] is a statistical technique used for classification in machine learning. The basic idea behind LDA is to find a linear combination of features that maximizes the separation between classes while minimizing the variation within each class. SVM [49] is a popular machine learning algorithm that can be used for classification and regression analysis. The basic idea behind SVM is to find the hyperplane that best separates the data points into different classes. SVM is particularly effective when the number of features in a dataset is large, as it can work well in high-dimensional spaces. SDA [50] is a variant of LDA and principal component analysis (PCA) whose goal is to find a subspace of the original feature space that maximizes the differences between classes. SDA is generally more effective than LDA when the number of features in a dataset is large and the dataset is high-dimensional.

#### 2.2.3. Image Feature Extraction

Image features include color features, texture features, shape features, and so on. In this study, 1 color feature, 4 texture features and 1 shape feature were selected for analysis. For the color feature, the color moment was selected. The color moment is a statistic used to describe image color features. For the texture features, the Gray Level Co-occurrence Matrix (GLCM) [51], Gray-Gradient Co-occurrence Matrix (GGCM) [52], Gray Level Difference Method (GLDM) [53], and Tamura texture [54] were selected. A GLCM describes the texture features of an image by calculating the gray level co-occurrence relationships between adjacent pixels in the image and counting the frequency of occurrence of these co-occurrence relationships in different directions and distances. A GGCM not only reflects the relationships between gray pixels, but also reflects the relationships between gradients, which can describe texture well. The GLDM is a statistic used to describe the texture characteristics of an image and can reflect the degree of difference between different gray levels. A variety of texture features can be calculated based on the GLDM. Tamura texture is a feature extraction method based on human visual perception that aims to simulate the human perception process of texture, including coarseness, contrast, directionality, linearity, regularity, and roughness. The shape feature selected for this study was the image moment. Image moments are mainly of two types: the original moment and the normalized moment. The specific features selected in this study are shown in Table 1.

### 2.3. Data Training and Model Evaluation

#### 2.3.1. Dataset Construction

The acquired hyperspectral data were extracted, and the background of the single-channel image in the hyperspectral image was removed using a threshold segmentation method to obtain a binary image, which was applied to the full-band spectrum to remove the background. The whole leaf was defined as a region of interest (ROI).

**Figure 4:**
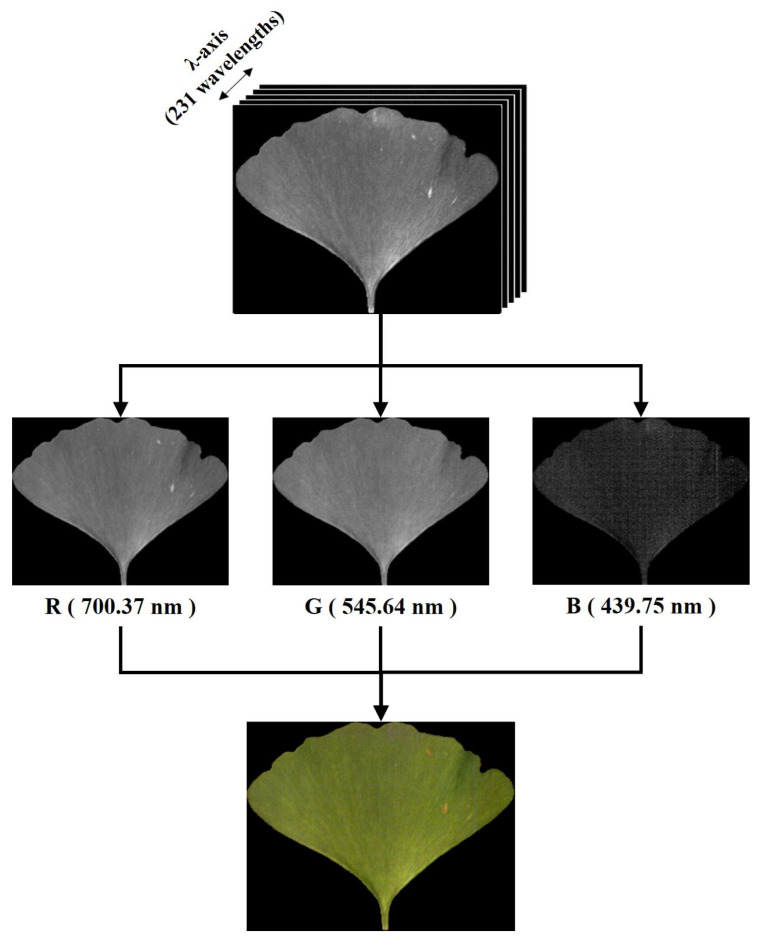
Method for synthesizing RGB images from hyperspectral imaging data.

This research included three datasets, namely, the RGB image dataset, spectral dataset, and image feature dataset. Considering the cost and convenience of subsequent technical application, RGB images were extracted from the hyperspectral data as the basic data of images dataset. The extraction process involved selecting the images whose bands were closest to red light (700 nm), green light (546 nm), and blue light (439 nm) in the hyperspectral data and synthesizing the images of the three channels. For the hyperspectral imager used in this experiment, the images were 700.37 nm, 545.64 nm, and 439.75 nm bands, as shown in Figure 4. The spectral dataset consisted of the average spectrum of a single ROI, and a total of 210 spectral signals were intercepted in the range of 437 to ~998 nm due to the presence of obvious noise signals at both ends of the spectral curve. The image feature dataset was extracted using the method in Section 2.2.3, with a total of 53 variables, including 1 color feature, 4 texture features, and 1 shape feature.

The total data from the Ginkgo leaves were divided into three parts according to proportion of the total sample size. Specifically, a training set, a validation set, and a test set were created in a ratio of 3:1:1 within a 4-fold cross-validation routine. The original leaves corresponding to different data in each set remained consistent.

Due to the limitation of the spectral resolution, the bands used for the RGB images extracted from the hyperspectral data were different from those utilized by commonly used visible light cameras. Therefore, whether this method could be applied to images captured by visible light cameras needed to be verified. *Ginkgo biloba* leaves were collected on 3 November 2022, and 1 August 2023 at the Zijingang Campus of Zhejiang University, and images were taken with a mobile phone. The phone used was a vivo X60 (Vivo Communication Technology Co. Ltd, Dongguan, Guangdong, China) with a resolution of 4000 × 3000 pixels. The ginkgo leaves were placed on a black absorbent cloth in a 4 × 3 arrangement. The distance between the phone and the leaves was 30 cm when capturing the images. Finally, 500 male images and 500 female images were selected for each of the two collections. Modeling was performed with calibration sets of different sizes (200, 400, 600, and 800). At the same time, the sizes of the validation and prediction sets were set at 200 and 1000 images, respectively.

#### 2.3.2. Modeling Setup

To train the synthesis of RGB images from the hyperspectral data, a pretrained model based on ImageNet was selected as the basis for all training plans. The optimizer was stochastic gradient descent (SGD) with a momentum of 0.9. The batch size was set to 64, and a scheduled learning rate was used. Initially, the learning rate was set to 0.01, and decreased ten times after every 30 epochs. The maximum number of training epochs was set to 100, and the training weights were stored and evaluated every epoch. The best weights trained on the evaluation dataset were kept for testing.

For the data from mobile phones, the optimizer, learning rate adjustment strategy, maximum training epoch, and model retention were the same as in the previous training, with the batch size set to 32.

For the one-dimensional data modeling, hyperparameter optimizations were chosen as follows. The covariance of the LDA method was set to full rank. The quadratic function was used as the kernel function for SVM; the kernel scale was automatic; the regularization parameter (C) was 1; and the multiclass method was One-vs-One. The number of SDA learners was set to 30, and the subspace dimension was set to half the number of the features.

The Windows 10 operating system was used in this study. The central processing unit (CPU) was an Intel Core i9-12900K, and the graphics processing unit (GPU) was an NVIDIA GeForce RTX 3090 Ti. All modeling processes were performed in MATLAB^®^ software (R2021b, MathWorks, Natick, MA, USA).

#### 2.3.3. Model Evaluation

It was critical to evaluate the model’s performance with appropriate indicators. Classification accuracy was used to evaluate the qualitative analysis models, and was calculated as the ratio of correctly classified samples to the total number of samples. Values closer to 100% indicated better performance.

## 3. Results

### 3.1. RGB Classification Results Based on Deep Learning

#### 3.1.1. Model Selection

In this study, ResNet101, Shufflenet, NASNet, Inception-v3, and Inception-ResNet-v2 models were built based on all leaf data in the green leaf and yellow leaf stages. The results are displayed in Table 2. For the prediction set, all models had overall accuracies greater than 85%, indicating that the deep learning network could capture the feature differences between male and female leaves to achieve effective differentiation. The ResNet101 model performed best, with an overall accuracy of 87.74%, followed by the NASNet and Inception-ResNet-v2 models with similar results (87.55% and 87.16%).

The purpose of extracting RGB images from hyperspectral data was to facilitate the subsequent technical application as much as possible, so the size and inference speed of the model were also indicators that needed to be measured. The inference speed of the NASNet model was too slow, and the real-time detection requirement was not realized when the processor performance was reduced. Meanwhile, Inception-ResNet-v2 had too many parameters, which increased the memory requirement. Taking these factors into consideration, the ResNet101 model was the most suitable model for this sex identification task in *Ginkgo biloba*.

#### 3.1.2. Background Expansion

**Figure 5:**
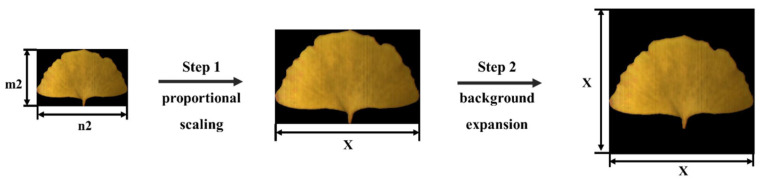
Proportional scaling–background expansion method workflow.

In addition to model selection, different image preprocessing methods also had a great impact on the accuracy of the classification. In order to improve the accuracy, a proportional scaling–background expansion method was proposed. The specific operation involved adjusting the size of the image proportionally until its long side reached a fixed value, filling a black background around the image, and forming a square image with the long side as the side length, as shown in Figure 5. The fixed value was set to 560 pixels in this study.

**Figure 6:**
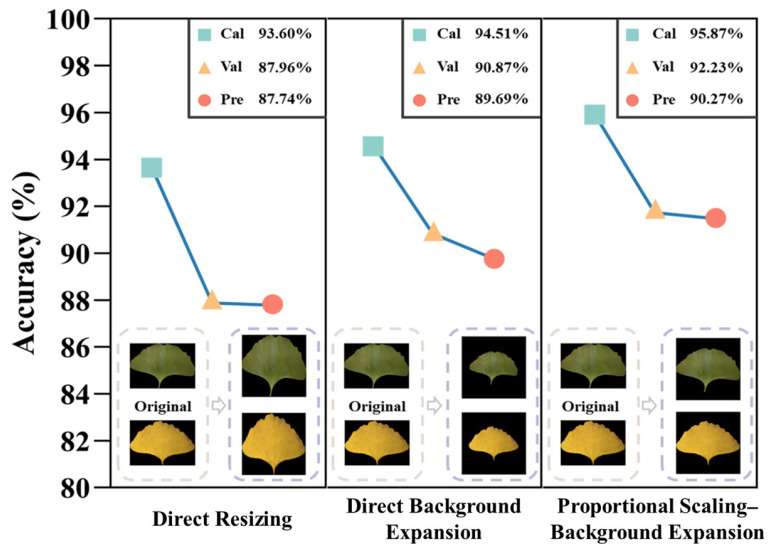
Modeling results of different preprocessing methods.

The proposed proportional scaling–background expansion method was compared with two other preprocessing methods. One of these methods was to directly resize the image to a square, and the other was to directly fill the black background without changing the size or shape of the image to form a square image with a fixed size. Here, the fixed size was also set to 560 pixels. The above data were modeled based on the ResNet101 model, and the results are presented in Figure 6. The proportional scaling–background expansion method achieved the best result. The accuracy on the prediction set was 90.27%, which was 2.53 and 0.58 percentage points higher than the accuracy obtained using the direct resizing and direct background expansion methods, respectively. This indicated that the proportional scaling–background expansion preprocessing method was effective.

#### 3.1.3. Verification Using Images Taken with Mobile Phones

Based on the data obtained using mobile phones, sex identification models were constructed using ResNet101. The pretrained model based on ImageNet and the optimal model in Section 3.1.2 (pretreated by proportional scaling–background expansion) were used as the basic parameters of the training model. As shown in Table 3, the accuracy of the optimal model on the prediction set reached 88.20%, which was similar to the accuracy of the previous model based on the RGB images extracted from the hyperspectral data. At the same time, it was observed that with a reduction in the number of calibration sets, the accuracy of the models on the prediction set decreased. However, the models trained with the optimal model from Section 3.1.2 had a lower rate of decline than those trained with the ImageNet-based pretrained model. Even when the modeling sets only contained 200 images, the model trained with our optimal model as the initial parameter still had 85.75% accuracy on the prediction set. Therefore, this indicated that the method of building a ResNet101 model through the proportional scaling–background expansion preprocessing operation was also applicable to the images captured using visible light cameras. Meanwhile, this study provides an effective pretrained model for completing *Ginkgo biloba* sex classification tasks based on RGB images, which could help achieve better results in subsequent migration and applications.

### 3.2. Classification Results Based on Spectral Information

#### 3.2.1. Spectral Feature

The optimal result of the model based only on RGB images was an accuracy of 90.27% on the prediction set, which left room for improvement on this binary classification task.

**Figure 7:**
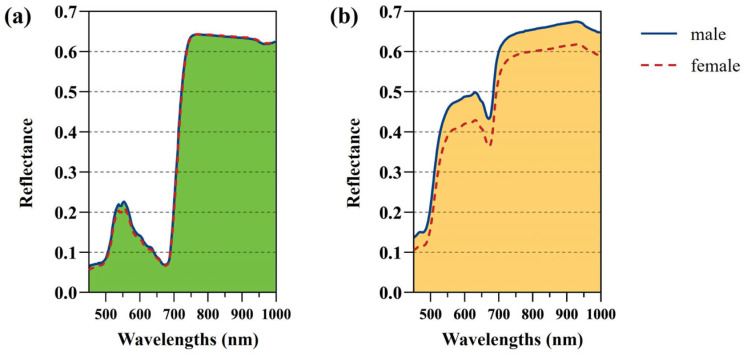
Spectra of the ginkgo leaves. (**a**) Average spectra of the green leaves. (**b**) Average spectra of the yellow leaves.

The spectral variation trend of all leaves in a single period was consistent, but the variation trends were different between two periods. According to the average spectral curves (Figure 7), the reflectance of the male leaves was significantly higher than that of the female leaves in the whole wavelength range in the yellow leaf stage, and the reflectance of the male leaves was slightly higher than that of female leaves in the ranges of 437 to ~500 nm and 520 to ~640 nm in the green leaf stage. Overall, the leaves of ginkgo trees of different sexes were different in part of the spectral interval, and it was feasible to distinguish the sexes of ginkgo using the spectral curve.

#### 3.2.2. Effects of Leaf Stage

Because of the differences in the spectral trends between the two periods of leaf sample collection, it could be assumed that single-period modeling might yield better results. Therefore, in this section, the results of two-period modeling and single-period modeling are compared. The results are presented in Table 4.

In general, the modeling results based on a single period were better than those based on two periods. For the green leaf stage, the results of SVM and SDA models based on the green leaf stage spectra were better than those based on the mixed spectra, and their accuracy on the prediction set was improved by 6.32 and 2.37 percentage points, respectively. For the yellow leaf stage, the results of LDA and SDA models based on the yellow leaf stage spectra were better than those based on the two-period spectra, and their accuracy on the prediction set was improved by 5.36 and 2.30 percentage points, respectively. At the same time, it should be noted that regardless of the modeling method, the results for the yellow leaf stage were better than those for the green leaf stage. These results were consistent with the differences reflected in the spectral curve. Finally, in the green leaf stage, the SVM and SDA models obtained optimal results with data from a single green leaf stage, and their accuracy on the prediction set was 87.35%. In the yellow leaf stage, the LDA model with data from a single yellow leaf stage obtained the optimal results, and its accuracy on the prediction set was 98.85%.

#### 3.2.3. Model Results Based on the Two-Stage Period-Predetermined Method

The models based on single-period data performed better. However, using these models required identifying the period before classification. Based on this, a two-stage Period-Predetermined (PP) method for *Ginkgo biloba* sex classification was proposed; i.e., period identification was performed first, and the corresponding model was then selected for sex classification according to the identification results. After period differentiation, the majority voting algorithm [56], where the final result was determined by the consensus of most models, could be combined with the PP method.

**Figure 8:**
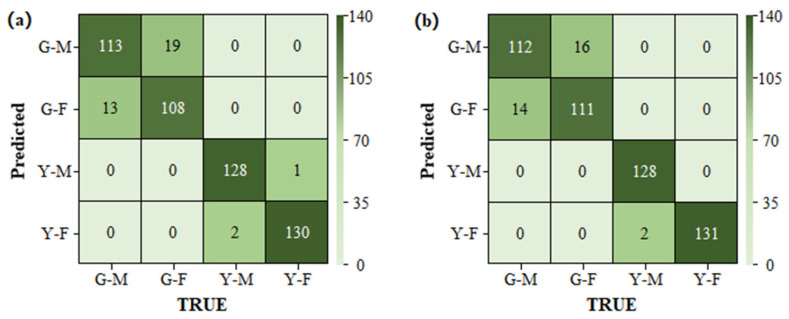
Confusion matrices for the prediction set. (**a**) The Period-Predetermined method alone. (**b**) The PP method combined with a majority voting decision. Note: G indicates the green leaf stage; Y indicates the yellow leaf stage; M indicates male; and F indicates female.

Due to the large difference between the spectra in the different periods, all commonly used classification methods could achieve accurate classification. In this study, the LDA method was selected. For the PP method, the SDA model based on single-period data was selected as the sex classification model for the green leaf stage, and the LDA model based on single-period data was selected for the yellow leaf stage. For the PP method combined with the majority voting algorithm, the SVM and SDA models based on the single-period data, and the LDA model based on the two-period data were selected as the sex classification models for the green leaf stage. The LDA and SDA models based on data from a single period, and the SDA model based on data from two periods were selected for the yellow leaf stage. The results of the PP method and of the PP method combined with a majority voting decision on the prediction set are shown in Figure 8.

The overall accuracy of the PP method combined with the majority voting decision was 93.77%, slightly higher than that without the introduction of majority voting. However, the introduction of majority voting involved multiple models, and the computational cost was greatly increased. Therefore, considering the portability of the subsequent algorithms, the PP method alone was considered to be a better method to balance accuracy and computation.

### 3.3. Classification Results Based on Fusion Information

#### 3.3.1. Results Based on Image Information

In order to fully utilize the hyperspectral information, the spectral information and image information were fused to improve classification accuracy. To reduce the computational complexity and modeling difficulty, the image features extracted from the original image were selected as inputs. In this study, a total of 53 variables were extracted, including 1 color feature (color moment), 4 texture features (GLCM, GGCM, GLDM, and Tamura texture), and 1 shape feature (image moment). To verify the quality of the extracted image features, the image features were first analyzed alone, and the results are shown in Table 5. The results show that the accuracies of the models based on the extracted image features were higher than 80% on the prediction set. This indicates that the extracted features could characterize the differences between ginkgo leaves of different sexes and were valid for this classification task.

#### 3.3.2. Results Based on Fused Data

**Figure 9:**
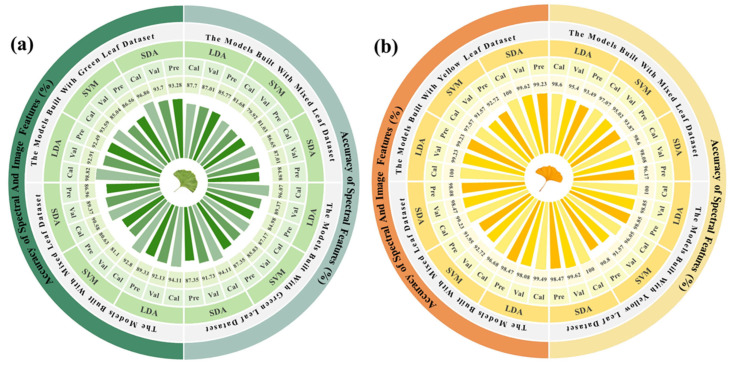
The results of the comparison between the fusion data and spectral data: (**a**) The green leaf stage. (**b**) The yellow leaf stage.

The fusion data contained 263 features, of which 210 were spectral features and 53 were image features. The above results show that there was a significant difference in the performances of the models built using spectral data from a single period or the mixed period. Therefore, this difference is also compared in this section. The modeling results based on the fusion data and a comparison with those based on the spectral data are shown in Figure 9.

Compared with the modeling results using the spectral data, model performance was improved using the fusion data, except for the SVM models based on the single-period data, which indicated that the fusion of spectral features and image features could contain more effective information, and was conducive to the task of *Ginkgo biloba* sex classification. At the same time, among the modeling results based on the fusion data, the models based on a single period were superior to the models based on two-period data, indicating that these features were significantly different in the different leaf growth periods. Finally, the optimal results were obtained using the SDA method to model the single-period fusion data, and the accuracy values for the prediction set were 93.28% for the green leaf stage and 99.23% for the yellow leaf stage, which were 5.93 and 0.38 percentage points higher than the optimal results based on spectral data, respectively.

#### 3.3.3. Model Results Based on the PP Method

**Figure 10:**
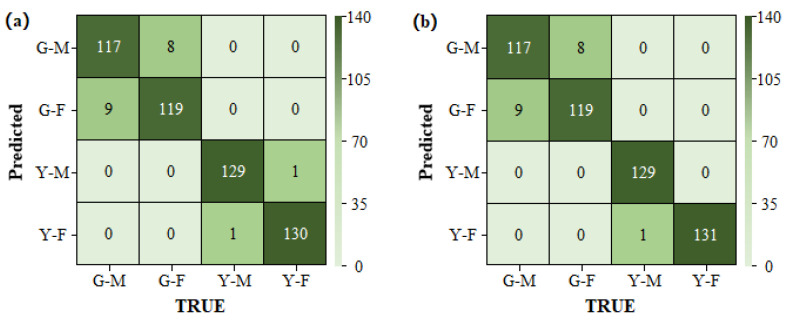
Confusion matrices for the prediction set. (**a**) The Period-Predetermined method alone and (**b**) The PP method combined with a majority voting decision. Note: G indicates the green leaf stage, Y indicates the yellow leaf stage, M indicates male, and F indicates female.

This section compares the improvement in model performance when the PP method was combined with a majority voting decision. For the PP method without a majority voting decision, the SDA model based on the data from the corresponding period was selected as the sex classification model. The basic models selected for the PP method combined with a majority voting decision were the SDA and LDA models based on data from a single period and the LDA model based on the data from the two periods for both the green and yellow leaf stages. The results for the prediction set are shown in Figure 10. The overall accuracy of the majority voting decision was 96.50%, which was only 0.2 percentage points higher than that of the PP method alone—an almost negligible difference. Therefore, the Period-Predetermined method alone, with its lower computational cost, was still considered to be the optimal algorithm.

## 4. Discussion

This study is the first to merge hyperspectral imaging and machine learning to classify the sexes of *Ginkgo biloba* L. using leaf data. A sex classification model for *Ginkgo biloba* was established using spectral information and image data. For RGB image data, the optimal detection result of 90.27% was achieved on the prediction set based on a ResNet101 model combined with a proportional scaling–background expansion method. Using one-dimensional spectral information, based on the fusion data of spectral information and image features combined with the two-stage PP method proposed in this study, the difference between ginkgo leaves in different developmental stages could be fully considered to achieve the efficient classification of male and female ginkgo trees. For the prediction set, the accuracy for the green leaf stage was 93.28%; the accuracy for the yellow leaf stage was 99.23%; and the overall accuracy reached 96.30%.

The main advantages of this classification method based on hyperspectral imaging technology were its ease of use, fast detection speed, and wide application range. In our study, models that utilized solely spectral data displayed superior recognition capabilities compared to those based on direct image recognition through deep learning techniques. This result shows the unique effectiveness of spectral data in identifying ginkgo sexes that may not be as apparent in standard RGB images. Moreover, integrating image features into our models led to an enhancement in accuracy. This improvement suggests a synergistic interaction between the spectral data and image features. By combining the strengths of both data types, our approach was able to capture a more comprehensive array of informative characteristics, resulting in more accurate classifications. This method could achieve identification using only leaves, and hardly any preprocessing work was required after leaf collection. Data acquisition for a single leaf could be completed in a few seconds, and the entire data processing sequence, including background removal, ROI extraction, data extraction, and model prediction, was automated. The complete process took less than one minute per leaf. At the same time, since the data used in the modeling included both the green and yellow leaf stages, the two states that *Ginkgo biloba* leaves exhibit throughout the year, the model could be applied to the sex identification of ginkgo during most of the year.

Two common methods for identifying male and female ginkgo based on molecular markers and physiological and biochemical characteristics were compared. Lee et al. [57] used a loop-mediated isothermal amplification (LAMP) method, which was simpler and faster than traditional PCR methods, to distinguish ginkgo sex, and only 10 ng of ginkgo DNA was required for detection. However, it is worth noting that although the amplification time of this method was shorter than that of traditional PCR methods, the entire amplification process still took 80 min. Fifteen male samples and fifteen female samples were used to verify the accuracy of the method. As a result, both the male and female samples had one prediction error, and the accuracy was 93.3%, which was lower than the result of this study. Like this study, Fu et al. [15] classified sex based on the different physiological and biochemical characteristics of male and female ginkgo, but, in contrast to this study, they adopted an electrochemical method. The leaves and petioles of ginkgo were selected as the target organs for analysis, and the electrocatalytic reduction of H_2_O_2_ by peroxidase was used as a probe to achieve rapid identification. The clustering accuracy of 25 male samples and 25 female samples was 100%. Although the accuracy of this study was high, grinding was required before testing, and the entire electrochemical identification process took more than 10 min. Therefore, when achieving a large number of detections is required, the method in this study will be more advantageous.

When promoting the application of this technology based on hyperspectral imaging and deep learning for Ginkgo sex classification, the following important factors need to be considered. Hyperspectral imaging and deep learning methods are computationally intensive. Implementing these techniques on a large scale would require significant computational power and storage capabilities. Advanced GPUs and high-performance computing systems are necessary to process and analyze the data efficiently. Furthermore, we will explore the way to integrate this method with existing agricultural and forestry management systems. This integration would involve developing user-friendly interfaces, data management systems, and decision-support tools that can utilize the classification results effectively. In summary, the method developed in this study shows significant promise for the classification of ginkgo sexes, and further research and development efforts should focus on optimizing these methods for practical use and developing solutions to overcome computational resources, scalability, cost, and system integration challenges.

## 5. Conclusions

In this study, the method of ginkgo sex classification based on hyperspectral imaging technology was first reported. This approach can collect data within a few seconds without any pretreatment of the samples, and the data processing can be realized automatically. We introduced the Period-Predetermined (PP) method to maximize the utility of the optimal models, achieving an overall prediction accuracy of 96.30%. This method identified the trees’ phenological period first. Then, the corresponding model was selected for sex classification according to the identification results. After the differentiation of period, the majority voting algorithm, in which the final result was the result of the majority of the models, could be combined with the PP method. In future research, we can focus on the differences in spectral values between juvenile seedlings and adult trees. By establishing the reliability of hyperspectral imaging across various developmental stages of plants, we aim to extend the application of this technology to accurately determine the sexes of plants in nurseries and of other dioecious species. This progression will enhance our understanding of spectral data’s role in sex identification and its potential scalability for broader agricultural and forestry applications.

## Figures and Tables

**Table 1:** Summary of feature selection.

Feature Type	Specific Feature	Number of Features	Details
Color features	Color moment	6	The mean values and standard deviations of the red, green, and blue channels.
Texture features	GLCM	16	Contrast, correlation, energy, and homogeneity at 0°, 45°, 90°, and 135°.
	GGCM	15	Small-gradient dominance, large-gradient dominance, uniformity of gray distribution, uneven gradient distribution, energy, gray mean, gray mean square error of gradient, correlation, gray entropy, gradient entropy, mixing entropy, inertia, and deficit moment.
	GLDM	4	The mean value, contrast, directional second moment and entropy.
	Tamura texture	5	Coarseness, contrast, directionality, linearity, and roughness
Shape features	Image moment	7	Geometric moments proposed by Hu [55].

**Table 2:** Sex identification results based on leaves of two stages.

Model	Accuracy (%)			Inference Speed (Files/s)	Parameter Quantity (M)
	Calibration Set	Validation Set	Prediction Set		
ResNet101	93.60	87.96	87.74	35.18	44.6
NASNet	92.64	87.57	87.55	9.68	5.3
Inception-ResNet-v2	93.22	86.21	87.16	55.29	55.9
Inception-v3	90.57	83.69	86.58	24.19	23.9
Shufflenet	90.89	84.66	85.41	65.50	1.4

**Table 3:** Sex identification results based on images taken with mobile phones.

Size of Calibration Set (Images)	Initial Parameter	Accuracy (%)		
		Calibration Set	Validation Set	Prediction Set
800	ImageNet	88.50	86.00	84.60
	Ours	95.25	89.00	88.20
600	ImageNet	88.17	82.00	80.80
	Ours	93.83	90.00	87.20
400	ImageNet	88.75	82.00	79.00
	Ours	92.00	88.00	86.20
200	ImageNet	91.50	78.00	77.10
	Ours	91.50	82.00	85.80

**Table 4:** Sex identification results based on spectra.

Period	Data	Method	Accuracy (%)		
			Calibration Set	Validation Set	Prediction Set
G	d	LDA	87.70	87.01	85.77
		SVM	81.68	79.92	81.03
		SDA	86.65	87.01	84.98
	s	LDA	96.07	89.37	84.98
		SVM	87.17	85.83	87.35
		SDA	94.11	91.73	87.35
Y	d	LDA	98.60	95.40	93.49
		SVM	97.07	95.02	93.87
		SDA	98.60	98.08	96.17
	s	LDA	100.00	98.85	98.85
		SVM	96.05	91.57	90.80
		SDA	100.00	99.62	98.47

Note: G indicates the green leaf stage, and Y indicates the yellow leaf stage; d indicates that the modeling data were the spectra of double-period leaves, and s indicates that the modeling data were the spectra of single-period leaves.

**Table 5:** Sex identification results based on image features.

Method	Accuracy (%)		
	Calibration Set	Validation Set	Prediction Set
LDA	84.04	83.07	80.58
SVM	93.22	84.24	83.69
SDA	91.28	82.65	81.52

## Data Availability

The data presented in this study are available on request from the corresponding author.
